# Supplementary material for: Effect of testing criteria for infectious disease surveillance: The case of COVID-19 in Norway
Source: PLoS One. 2024 Aug 15;19(8):e0308978. doi: 10.1371/journal.pone.0308978 (PMC11326602; doi:10.1371/journal.pone.0308978)
Supplement: S1 Text — Additional supplementary results and methods. (PDF) [file pone.0308978.s001.pdf]

Supporting information appendix for: Effect of testing criteria for infectious disease surveillance: the case of COVID-19 in Norway

Circular block bootstrap

We estimate the uncertainty of the fitted models by block bootstrap (Künsch 1989). This is a resampling method which can be used for time dependent data. Specifically, we use a circular block bootstrap (Politis & Romano 1993) to split the data into different blocks as following:

- We first draw a start index for the blocks.
- Starting from the start index, we draw one block at the time by drawing the block length as a random draw from a uniform integer distribution between a fixed minimal and maximal block length.
- We assume circularity, such that the first observation follows the last observation of the data.

We then draw the bootstrap sample by drawing blocks with replacement until we obtain as many data points as originally in the data (here 274 days). The length of the last block is shortened if needed, such that we obtain exactly 274 days. We draw a new block distribution for each bootstrap sample (i.e. repeat the points above). If the bootstrap sample does not contain any public holidays, we replace the sample by a new bootstrap sample. Altogether we draw 500 bootstrap samples. The same bootstrap sample is used for all the models fitted in the paper. The regression models are fitted for each bootstrap sample. GAM has an inbuilt, automatic method for estimating the smoothing parameter for each spline. We let these be equal to the fit on the original data set for all the bootstrap fits.

We study the effect of the block lengths for one of the regression models, where the probability of testing positive is modelled as a function of hospital incidence, time, and weekday. We report the estimated standard deviation for the parametric estimates and assess how these vary with different block lengths (Table S1). We note that the standard deviations seem to be relatively stable for the different block lengths, and chose to use a minimal block length of 7 days and maximal block length of 14 days for all the models in the paper.

	Saturday/Sunday	Monday	Bank holiday
Minimum 3, maximum 5	0.038	0.036	0.11
Minimum 7, maximum 14	0.03	0.031	0.12
Minimum 14, maximum 28	0.031	0.033	0.13

Table S1. Estimated standard deviations for different block lengths for the parametric coefficients of the model.

References

Kunsch, H. R. (1989). The jackknife and the bootstrap for general stationary observations. The annals of Statistics, 1217-1241.

Politis, D. N., & Romano, J. P. (1993). Nonparametric resampling for homogeneous strong mixing random fields. Journal of Multivariate Analysis, 47(2), 301-328.
