# Supplementary material for: Effect of testing criteria for infectious disease surveillance: The case of COVID-19 in Norway
Source: PLoS One. 2024 Aug 15;19(8):e0308978. doi: 10.1371/journal.pone.0308978 (PMC11326602; doi:10.1371/journal.pone.0308978)
Supplement: Attachment — Submitted filename: Response_to_reviewers.pdf [file pone.0308978.s002.pdf]

Dear Editor,

Thank you for the positive response to our manuscript. We are thankful for the good suggestions from the referees which have improved our manuscript. We have tried to answer the comments to the best of our ability, and we hope that we have answered the comments satisfactorily. The changes to the manuscript text are highlighted with track changes. We also provide a clean version. Below, we provide a point-by-point response to the reviewer comments, with our response in red.

Best regards

Solveig Engebretsen and Magne Aldrin

Reviewer #1: Review of "Effect of testing criteria for infectious disease surveillance: the case of COVID-19 in Norway"

This paper is a significant contribution which discusses how PCR testing of suspected cases varies due to testing policy criteria. To clarify its contribution, however, I recommend a major revision of the manuscript.

In the introduction, I suggest the authors provide a clearer context for this study. This could involve a more explicit comparison to what we currently understand about the role of PCR testing and its surveillance impact during COVID-19. This clarity will help readers better understand how Norway's approach compares and what unique findings this study offers. The main finding stressed by the authors is that "smaller changes in testing criteria do not seem to have large, abrupt consequences for the disease surveillance, larger changes like the introduction and massive use of self-tests makes the test data less useful for surveillance." I think the authors need to further develop this argument in the introduction, and better explain how they arrive at this conclusion especially since the authors are only analyzing PCR testing data, even if considering changes in antigen and PCR testing policies.

We have included more background on infectious disease surveillance and use of confirmed cases during the pandemic for surveillance in general and modelling in particular. Moreover, we have argued why testing criteria need to be considered in terms of the observation model used when performing inference in infectious disease models. The added paragraphs read

The different surveillance data sources have different advantages and limitations, in terms of for example accuracy, representativity, noise, timeliness, acceptance, and cost and resource demands [4, 5]. It is therefore common to combine evidence from multiple surveillance data sources. Specifically, combining evidence in different data sources was also recommended by WHO for COVID-19 surveillance purposes [6]. Another example is the routine influenza surveillance system in Norway which includes, among other sources, data on the number of general practitioner visits related to influenza, sentinel surveillance and laboratory-based surveillance of patients with acute respiratory infection, seroprevalence studies, and hospital prevalence [7]. Laboratory-based surveillance and seroprevalence studies are in general less noisy than data on influenza-like illness diagnoses from general practitioners, but more delayed and costly.

During the COVID-19 pandemic, data on confirmed cases were used for surveillance and modelling purposes in many countries, for example when estimating reproduction numbers (see for example [8, 9, 10, 11]). Specifically, confirmed cases were included in the WHO's global COVID-19 surveillance system, in addition to data on e.g. the total number tested, deaths and hospitalisations due to COVID-19 [12]. Moreover, WHO's guidance for public health surveillance of COVID-19 states that data on number of confirmed cases from laboratory tests should be collected as they are useful for early warning of changes in epidemiological patterns and to monitor trends in infection [6]. During the COVID-19 pandemic, multiple countries also used environmental surveillance, in particular wastewater surveillance. Such environmental surveillance has the advantage of not being affected by health care seeking behaviour, disease severity, or testing criteria/capacity [13]. Norway also used wastewater surveillance from June 2022 [10]. A systematic review on the use of environmental surveillance during COVID-19 concludes that wastewater surveillance could be used as an early warning signal and hence complement clinical surveillance of SARS-CoV-2 [14], but that the cost-benefit needs to be taken into account. Wastewater surveillance could also contribute to early detection of new variants. As noted by WHO, laboratory-confirmed cases remain the primary source of evidence on incidence of infection [13].

The Norwegian Institute of Public Health used both hospital incidence and confirmed cases for inference in the mathematical models of COVID-19 spread and for estimating reproduction numbers [15, 16]. Hospital incidence is a more robust surveillance source than test data, as it does not depend on testing criteria and testing capacity. Hospital incidence is also less sensitive to health seeking behaviour and self-selection. In addition, one of the main uses of mathematical modelling for real-time situational awareness is to predict hospitalisations, and hence it is important to calibrate to hospital data. There are, however, fewer observations in the hospital data than in the test data, they are more delayed, and they generally represent a population at higher risk than the community at large. Moreover, it is problematic to use hospital case data for surveillance if the hospital capacity is reached. To our knowledge, this was not a problem during the pandemic in Norway. Hence, one of the largest limitations with using hospital incidence data alone in infectious disease surveillance is that it takes more time before a change in transmission is visible in the hospital data than in the test data. It is therefore important to combine information from both data sources when calibrating mathematical disease spread models and in surveillance. Though data on confirmed cases from laboratory tests are subject to under reporting due to e.g. health care seeking behaviour, they can still be used relatively straightforward to monitor trends as long as the under reporting stays constant [5]. Nonetheless, it is difficult to use test data in periods where the testing strategy is unstable, that is, when the meaning of a positive test changes over time. If the meaning of a positive test changes, this will potentially have consequences for surveillance and estimated reproduction numbers [17]. Changes in testing criteria may lead to conflicting information in different data sources used for surveillance and/or for inference in mathematical models. If the changes in testing criteria have a practically significant effect on the outcomes of the models, then a complex observation model for the test data would be necessary. This is a nontrivial inference problem and will likely be subject to identifiability issues in a complex observation model unless the parameters are assumed fixed or given informative priors. In Norway, the modelling team from the Norwegian Institute of Public Health handled this in part by incorporating the total number of tests in a relatively simple observation model, as a proxy for changes in testing criteria and testing capacity. The test data were not used for model inference in the early period when testing had not stabilised. Moreover, a seven days rolling mean of the test data was used in the calibration, in order to take into account potential day-of-the-week effects [15]. Using the number of tests to estimate the reproduction number for COVID-19 was also done in for example [18, 19], and another approach to an observation model which takes into account changes in testing criteria can be found in [20].

I am not clear on the research design time period of the study. The main objective of the study

seems to be to use PCR test data from April 1, 2020 to 2022 to evaluate how changes in antigen and PCR testing policies affected the demand for PCR testing and positivity rates when antigen tests were introduced. However, on line 111 the authors write “We fitted three generalised additive regression models (GAM) [32] to the test data for 2020.” This is similar in the abstract which states “The regression analysis focuses on the time period until 2021, i.e. before Norway started 16 vaccination.” These sentences suggest that the cutoff is December 31, 2020. In fact, this is not the case as the figures show data up until 2022. The authors should provide precise dates and the number of weeks for the start and end date of the study.

Under the «Methods» section we now specify further the time periods used in the different parts of the paper. Specifically, we have added the following paragraph:

We analysed the data using two different approaches. The first was a purely explorative data analysis, where we inspected the data from the whole period available, that is, from April 1, 2020 to November 13, 2022. In addition, we fitted regression models to the daily test data from April 1, 2020 to January 1, 2021 (in total 274 days, one day missing). We chose to limit the regression analysis to this period only, as it was not cluttered by larger known changes in variant and by vaccination.

The study's objectives should be stated more clearly. The authors describe changes in PCR and antigen/lateral flow testing policies. Lateral flow tests were available in June/July 2021 and mass testing in August 2021. Testing criteria were considerably loosened in 2022. In other words, the research analyzes PCR outcomes, but changes in antigen and PCR testing policies.
Since this paper claims that its findings can be used to inform surveillance, it would be important to explain why antigen test results were used in the analysis combined with the PCR test results that were analyzed. What reporting systems were required for positivity notification for antigen and PCR specimens in Norway? How were the PCR test results obtained by the authors? Why wasn’t there research undertaken to explore laboratory capacity and scaling up over the period? Does Norway use sentinel surveillance for severe respiratory infections, and what does this data suggest compared to the overall volume and positivity rates of PCR testing in the general population?

In our study, we only use the publicly available number of confirmed cases made available from the Norwegian Institute of Public Health. These results were obtained by downloading them from the github data repository, as explained under the Data section. We do not have access to data on the number and results of self-tests, and such data were not systematically collected in Norway. As we do not use the self-tests in this study and only the PCR tests, we believe that it is not within the scope of our study to go into details on the reporting systems for self-tests in Norway. However, they were never systematically reported and recorded, particularly not the negative self-tests. We have added this information in the manuscript. Moreover, we have included more information in general on surveillance of infectious diseases, and we also briefly mention sentinel surveillance for SARI/influenza. However, we do not have access to these data and hence cannot compare them to the COVID-19 test data. The added paragraph on surveillance in general (also repeated in point 1) reads:

The different surveillance data sources have different advantages and limitations, in terms of for example accuracy, representativity, noise, timeliness, acceptance, and cost and resource demands [4, 5]. It is therefore common to combine evidence from multiple surveillance data sources. Specifically, combining evidence in different data sources was also recommended by WHO for COVID-19 surveillance purposes [6]. Another example is the routine influenza surveillance system in Norway which includes, among other sources, data on the number of general practitioner visits related to influenza, sentinel surveillance and laboratory-based surveillance of patients with acute respiratory infection, seroprevalence studies, and hospital prevalence [7]. Laboratory-based surveillance and

seroprevalence studies are in general less noisy than data on influenza-like illness diagnoses from general practitioners, but more delayed and costly.

With respect to methods, the authors could better explain why the GAM is the preferred method to analyze how testing changed over time due to changes in testing policies. Other studies have used event history analysis or structural breaks to identify how testing criteria impacted the total and positivity rates for testing, for example. As I understand this method, it has the advantage of allowing us to study an increasing growth trend considering seasonal trends. However, I think that the models as described suffer from omitted variable bias as the major policy announcements that add ripples to an otherwise smooth trend are not included in the specifications. Since these events are not included, the GAM might mistake nonlinear growth to be persistent occurrences and their effects would be erroneously propagated.

We have tried different regression models to study the effect over time. It is difficult to interpret the results from models with structural breaks (regression models where we allow for a change in level on different dates related to changes in testing policies) as we cannot separate the effect of changes on specific days from gradual changes over time due to other, unknown factors. Moreover, the effect of changes in testing criteria are likely gradual, and similarly changes in testing capacity are also likely gradual. We have therefore concluded that a smoothing spline in time was most appropriate for our study. Major policy announcements would be captured in the smoothing spline, though they might be smoothed. The smoothing parameter is selected through the in-built default smoothing parameter selection in the R-package mgcv. The general experience with this package is that the resulting effects are too volatile, i.e. not smooth enough, though this would of course not be true for an underlying truth containing discrete jumps. We have added a comment on the fact that we have also tried models with sudden jumps in the Methods section:

Note that we also tried out models which allowed for sudden jumps in accordance with the changes in testing criteria/policy. However, it turned out that the changes in number of tests and positive tests in 2020 were gradual and did not occur as sudden jumps.

Hospitalization incidence is justified as a “proxy” for the level of “infectiousness.” I am not sure about this justification, and I think it needs to be better explained. Why don’t the authors use the lag of the dependent variable as this would also capture infectiousness?

One of the key objectives of the study is to analyse the changes in testing criteria/capacity over time. This effect should ideally be adjusted for the infection level. We therefore adjust for the hospital incidence. We cannot include the lag of the dependent variable to capture infectiousness through for example an autoregressive model, as this would not allow us to study changes over time. This approach could, however, have been used if we were only interested in the weekday effects.

We have added the following to the discussion:

We adjusted for the hospital incidence as a proxy for the infection level in the population. This is unproblematic as long as the hospital incidence is a constant proportion of the number of infected individuals. However, this would not be the case if there were changes in viral variants/properties such that the severity of infected cases changed. If the hospital capacity is reached, then the hospital incidence data need to be treated as censored data, and that would have to be taken into account in the analysis. To our knowledge, the hospital capacity was not reached in Norway during the pandemic.

I am not sure why the authors use the index of i and not t when they report the models that will be estimated. It would be important to explain if this is weekly or daily data and the total number of time points.

To make this clearer, we have changed the index from i to d, where d represents day. We also specify that the regression analysis is based on 274 days of data.

Reviewer #2: I suppose my primary concern is with the entire premise of the paper, which is that changes in test criteria and self-selection for testing of COVID-19 (similar to any other infectious disease) will result in various biases in determined incidence of infection. I would propose that this is pretty obvious and a foregone conclusion, and one of the reasons for which, in many places around the world, various researchers have implemented water-based surveillance (and other more comprehensive, less-biased methods) of attempting to measure the true incidence of SARS-CoV-2 in communities.

Though there are other data sources for surveillance which are not subjected to self-selection, number of confirmed cases are considered useful for infectious disease surveillance to monitor trends. If the underreporting is constant (which may be a reasonable assumption under constant testing criteria), then it is relatively straightforward to use these data to monitor changes in e.g. reproduction numbers. We have included references to guidelines for COVID-19 surveillance stating use of confirmed cases for surveillance. We have also included examples of uses of test data during COVID-19, with examples of how changes in testing capacity were handled in the mathematical modelling for Norway and other references. In addition, we have included some additional background on surveillance of infectious diseases in general. We have also added references to environmental surveillance like wastewater surveillance for COVID-19. In general, the opinion on wastewater surveillance is that they represent a valuable complementary surveillance data source, which can be used *in addition* to clinical surveillance data sources. In particular, it is difficult to map from wastewater surveillance data to number of infections due to e.g. the large variation in viral load between individuals (see e.g. https://www.who.int/publications/i/item/WHO-HEP-ECH-WSH-2022.1). In addition, the validation of wastewater surveillance in the literature is through comparing these to clinical surveillance data sources, where the aim is to be able to explain the variation in these clinical surveillance data, like time series of hospitalisations and confirmed cases. As data on confirmed cases are collected also for other purposes than surveillance, it is highly unlikely that such readily available data will be fully disregarded in the surveillance, and hence this study contributes to understanding and quantifying the limitations of these data. The relevant sections in the introduction (added or modified from the previous version) are:

Confirmation of infection by testing has two main purposes: 1) finding out who is infectious in order to prevent spreading of the disease, and 2) surveillance, which is the focus of this study. There are many alternative data sources which can be used for surveillance of infectious diseases. The different surveillance data sources have different advantages and limitations, in terms of for example accuracy, representativity, noise, timeliness, acceptance, and cost and resource demands [4, 5]. It is therefore common to combine evidence from multiple surveillance data sources. Specifically, combining evidence in different data sources was also recommended by WHO for COVID-19 surveillance purposes [6]. Another example is the routine influenza surveillance system in Norway which includes, among other sources, data on the number of general practitioner visits related to influenza, sentinel surveillance and laboratory-based surveillance of patients with acute respiratory infection, seroprevalence studies, and hospital prevalence [7]. Laboratory-based surveillance and seroprevalence studies are in general less noisy than data on influenza-like illness diagnoses from general practitioners, but more delayed and costly.

During the COVID-19 pandemic, data on confirmed cases were used for surveillance and modelling purposes in many countries, for example when estimating reproduction numbers (see for example [8, 9, 10, 11]). Specifically, confirmed cases were included in the WHO's global COVID-19 surveillance system, in addition to data on e.g. the total number tested, deaths and hospitalisations due to COVID-19 [12]. Moreover, WHO's guidance for public health surveillance of COVID-19 states that data on number of confirmed cases from laboratory tests should be collected as they are useful for early warning of changes in epidemiological patterns and to monitor trends in infection [6]. During the COVID-19 pandemic, multiple countries also used environmental surveillance, in particular wastewater surveillance. Such environmental surveillance has the advantage of not being affected by health care seeking behaviour, disease severity, or testing criteria/capacity [13]. Norway also used wastewater surveillance from June 2022 [10]. A systematic review on the use of environmental surveillance during COVID-19 concludes that wastewater surveillance could be used as an early warning signal and hence complement clinical surveillance of SARS-CoV-2 [14], but that the cost-benefit needs to be taken into account. Wastewater surveillance could also contribute to early detection of new variants. As noted by WHO, laboratory-confirmed cases remain the primary source of evidence on incidence of infection [13].

The Norwegian Institute of Public Health used both hospital incidence and confirmed cases for inference in the mathematical models of COVID-19 spread and for estimating reproduction numbers [15, 16]. Hospital incidence is a more robust surveillance source than test data, as it does not depend on testing criteria and testing capacity. Hospital incidence is also less sensitive to health seeking behaviour and self-selection. In addition, one of the main uses of mathematical modelling for real-time situational awareness is to predict hospitalisations, and hence it is important to calibrate to hospital data. There are, however, fewer observations in the hospital data than in the test data, they are more delayed, and they generally represent a population at higher risk than the community at large. Moreover, it is problematic to use hospital case data for surveillance if the hospital capacity is reached. To our knowledge, this was not a problem during the pandemic in Norway. Hence, one of the largest limitations with using hospital incidence data alone in infectious disease surveillance is that it takes more time before a change in transmission is visible in the hospital data than in the test data. It is therefore important to combine information from both data sources when calibrating mathematical disease spread models and in surveillance. Though data on confirmed cases from laboratory tests are subject to under reporting due to e.g. health care seeking behaviour, they can still be used relatively straightforward to monitor trends as long as the under reporting stays constant [5]. Nonetheless, it is difficult to use test data in periods where the testing strategy is unstable, that is, when the meaning of a positive test changes over time. If the meaning of a positive test changes, this will potentially have consequences for surveillance and estimated reproduction numbers [17]. Changes in testing criteria may lead to conflicting information in different data sources used for surveillance and/or for inference in mathematical models. If the changes in testing criteria have a practically significant effect on the outcomes of the models, then a complex observation model for the test data would be necessary. This is a nontrivial inference problem and will likely be subject to identifiability issues in a complex observation model unless the parameters are assumed fixed or given informative priors. In Norway, the modelling team from the Norwegian Institute of Public Health handled this in part by incorporating the total number of tests in a relatively simple observation model, as a proxy for changes in testing criteria and testing capacity. The test data were not used for model inference in the early period when testing had not stabilised. Moreover, a seven days rolling mean of the test data was used in the calibration, in order to take into account potential day-of-the-week effects [15]. Using the number of tests to estimate the reproduction number for COVID-19 was also done in for example [18, 19], and another approach to an observation model which takes into account changes in testing criteria can be found in [20].

I would greatly appreciate if the following questions/concerns could also please be addressed:
1. What was the thought process behind choosing "time" and "weekday" as the explanatory variables in your regression model? Why not consider age, gender, socioeconomic status and other potential explanatory variables for more or less frequent testing and/or test positivity?

«Time» was chosen as an explanatory variable as the study is mainly interested in addressing the effect of changes over time. Weekday was chosen as we suspected it to be an important variable for both testing capacity and testing criteria, and we were interested in quantifying the effect of day-of-the-week. It turns out to be an important variable to explain testing heterogeneity, and an interesting variable for understanding test seeking behaviour. In addition, as long as the model has a daily resolution, it is not only of interest but also necessary to adjust for day-of-the-week since it affects the test data. If the main aim of the study had been to understand test seeking behaviour in general, then it would be important to include different demographic variables like age and socioeconomic status. Though such analyses are also important and interesting, they are outside the scope of this study. We are interested in studying changes over time, and hence such covariates would mainly be relevant for our study if we believed the differences in test seeking behaviour among different groups to be confounded with time. It is, nonetheless, not likely that such variables would be included in the observation model when performing inference in an infectious disease model, as it would require age-, gender- and sociodemographic-specific test seeking behaviour parameters, which would be infeasible. Moreover, in this study we have chosen to use publicly available data, and hence we did not have access to any disaggregations.

2. I agree that hospital incidence provides more robust surveillance data than community test data in many ways but is laden with its own issues - primarily that it is biased towards more ill, more severe cases, and not necessarily reflective of what is happening in the general population in the community. I would argue that even in the hospital setting there still dependence on testing criteria, however, and that many hospitals may still have a limited capacity depending on available resources.

We agree that hospital incidence data have other limitations than their limited timeliness, and we have included a comment on how they are biased towards high-risk individuals. This is one of the reasons why we argue that it is important to calibrate to multiple data sources. Importantly, the hospital capacity was never reached in Norway during the pandemic. We have commented on the fact that it is problematic to use hospital data if the capacity is reached. Another reason why it is important to calibrate to hospital data is that one of the main uses of the mathematical model is to predict hospitalisations. We have also added a comment on this. The added/modified sentences in the manuscript are

Hospital incidence is also less sensitive to health seeking behaviour and self-selection. In addition, one of the main uses of mathematical modelling for real-time situational awareness is to predict hospitalisations, and hence it is important to calibrate to hospital data. There are, however, fewer observations in the hospital data than in the test data, they are more delayed, and they generally represent a population at higher risk than the community at large. Moreover, it is problematic to use hospital case data for surveillance if the hospital capacity is reached. To our knowledge, this was not a problem during the pandemic in Norway.

3. I can certainly see that in Norway, similar to many countries, there was variable availability of different testing available for different groups over time, as well as fluctuations in restrictions. It is not surprising that the number of positive PCR tests and the denominator of total PCR tests

completed also varied over time, depending on the testing protocol and COVID-19 restrictions in place.

As we also wrote in the reply to point 1, confirmed cases are widely used in infectious disease surveillance and during emerging outbreaks. Hence, it is important to understand the limitations of such data. In the study, we are interested in studying how the proportion of positive tests and number of tests changed over time (i.e. when testing criteria changed), in order to better understand how one can use and interpret the test data.

4. How was COVID-19 hospital incidence determined? Was this based on a positive test alone, clinical criteria alone or a combination of both? This is not clearly outlined in your Materials and Methods section.

The documentation of the data explains that this is the daily number of new patients admitted to hospital with COVID-19 as the main cause (as stated also in our manuscript). It does not specify whether these are confirmed COVID-19 cases. These data were publicly available during the pandemic, and official numbers reported weekly by the Norwegian Institute of Public Health. We have added this specification to the manuscript under Data:

These data were publicly available and reported weekly by the Norwegian Institute of Public Health during the pandemic.

5. Your generalized additive regression models appear to be well conducted; however, I am again curious in respect to your choice of time and weekday as your only explanatory variables? Why were demographics not considered as potential explanatory variables?

We refer to our answer to this question in point 1.

6. The use of hospital incidence as a proxy for infection level in the population is reasonable (and was an approach taken in many places), however, this does have its problems and there should be some discussion around the shortcomings of this assumption. Please address this.

We have added comments on properties of the different surveillance data sources in the manuscript and refer to our answer to point 2. In addition, we have added the following reflections to the discussion section:

We adjusted for the hospital incidence as a proxy for the infection level in the population. This is unproblematic as long as the hospital incidence is a constant proportion of the number of infected individuals. However, this would not be the case if there were changes in viral variants/properties such that the severity of infected cases changed. If the hospital capacity is reached, then the hospital incidence data need to be treated as censored data, and that would have to be taken into account in the analysis. To our knowledge, the hospital capacity was not reached in Norway during the pandemic.

7. I am confused by the statement that on "May 27, 2021 Norway opened up to testing without

suspicion of infection and mass testing, which seems to coincide with a decrease in both the total number of tests and proportion of positive tests." How did the denominator of total number of tests decrease if mass testing was being conducted - even without suspicion of infection?

We agree that this is not as expected. However, this is a purely explorative analysis, and hence there may be other reasons why the number of tests decreased. For example, this period in the plot covers the summer when there is in general less transmission. We have changed the phrasing to avoid confusion. The comment in the manuscript now reads:

On May 27, 2021 Norway opened up to testing without suspicion of infection and mass testing. Not surprisingly, the proportion of positive tests seemed to decrease. More surprisingly, the total number of tests also seemed to decrease, however note that this is purely an exploratory analysis, and we do not adjust for e.g. the decreased infection level in summer.

8. I do agree with your final conclusion that regular testing of a random (voluntary) sample of the population may have been helpful in allowing for better community surveillance of COVID-19, as stated. However, personally, I believe that there are potentially better, more comprehensive and less-biased ways to measure the incidence of COVID-19 in a community locale or region (such as waste-water based surveillance, etc).

We have added a comment that wastewater surveillance would be another alternative. As also noted in the answer to point 1, it is however nontrivial to map from viral load in wastewater to incidence in the population. Wastewater surveillance would likely have a higher sensitivity than testing a random sample of the population if the disease incidence is low, and we have also included a comment on this. The added sentences in the discussion are

Other alternatives would be to implement wastewater surveillance to complement the clinical surveillance [10]. The value of environmental surveillance is especially high when clinical testing is limited or the disease incidence is low [13].

9. In conclusion, you are quite correct in that we must be clear about the purpose of our testing in any given situation--whether it is to identify cases and stop transmission or try to accurately measure incidence or prevalence of an infectious disease. I am not certain how many countries and regions have necessarily used outpatient (community) testing as a surrogate measure for the latter, as opposed to the former, especially considering changes in testing protocols and restrictions over time.

We believe that as these data are readily available, they will always be used also as a low-cost surveillance data source. To clarify the use of data on confirmed cases as a surveillance data source, we have included references, for example to WHO's surveillance guidelines for COVID-19. We have also included references to mathematical modelling work from many different countries utilising confirmed cases to perform inference in their models. These models vary in how they take into account changes in testing criteria, but most contain simple observation models for the test data, as more complex observation models are often unfeasible, and instead include comments on these choices in their discussion sections. We repeat the relevant modified/added sentences here, which were also copied in on the answer to point 1:

During the COVID-19 pandemic, data on confirmed cases were used for surveillance and modelling purposes in many countries, for example when estimating reproduction numbers (see for example [8, 9, 10, 11]). Specifically, confirmed cases were included in the WHO's global COVID-19 surveillance system, in addition to data on e.g. the total number tested, deaths and hospitalisations due to COVID-19 [12]. Moreover, WHO's guidance for public health surveillance of COVID-19 states that data on number of confirmed cases from laboratory tests should be collected as they are useful for early warning of changes in epidemiological patterns and to monitor trends in infection [6].

and

Changes in testing criteria may lead to conflicting information in different data sources used for surveillance and/or for inference in mathematical models. If the changes in testing criteria have a practically significant effect on the outcomes of the models, then a complex observation model for the test data would be necessary. This is a nontrivial inference problem and will likely be subject to identifiability issues in a complex observation model unless the parameters are assumed fixed or given informative priors. In Norway, the modelling team from the Norwegian Institute of Public Health handled this in part by incorporating the total number of tests in a relatively simple observation model, as a proxy for changes in testing criteria and testing capacity. The test data were not used for model inference in the early period when testing had not stabilised. Moreover, a seven days rolling mean of the test data was used in the calibration, in order to take into account potential day-of-the-week effects [15]. Using the number of tests to estimate the reproduction number for COVID-19 was also done in for example [18, 19], and another approach to an observation model which takes into account changes in testing criteria can be found in [20].
